# Multi-targeting of functional cysteines in multiple conserved SARS-CoV-2 domains by clinically safe Zn-ejectors†

**DOI:** 10.1039/d0sc02646h

**Published:** 2020-09-01

**Authors:** Karen Sargsyan, Chien-Chu Lin, Ting Chen, Cédric Grauffel, Yi-Ping Chen, Wei-Zen Yang, Hanna S. Yuan, Carmay Lim

**Affiliations:** Institute of Biomedical Sciences, Academia Sinica Taipei 115 Taiwan carmay@gate.sinica.edu.tw; Institute of Molecular Biology, Academia Sinica Taipei 115 Taiwan hanna@sinica.edu.tw; Department of Chemistry, National Tsing Hua University Hsinchu 300 Taiwan

## Abstract

We present a near-term treatment strategy to tackle pandemic outbreaks of coronaviruses with no specific drugs/vaccines by combining evolutionary and physical principles to identify conserved viral domains containing druggable Zn-sites that can be targeted by clinically safe Zn-ejecting compounds. By applying this strategy to SARS-CoV-2 polyprotein-1ab, we predicted multiple labile Zn-sites in papain-like cysteine protease (PL^pro^), nsp10 transcription factor, and nsp13 helicase. These are attractive drug targets because they are highly conserved among coronaviruses and play vital structural/catalytic roles in viral proteins indispensable for virus replication. We show that five Zn-ejectors can release Zn^2+^ from PL^pro^ and nsp10, and clinically-safe disulfiram and ebselen can not only covalently bind to the Zn-bound cysteines in both proteins, but also inhibit PL^pro^ protease. We propose combining disulfiram/ebselen with broad-spectrum antivirals/drugs to target different conserved domains acting at various stages of the virus life cycle to synergistically inhibit SARS-CoV-2 replication and reduce the emergence of drug resistance.

## Abstract 2

By combining evolutionary and physico-chemical principles, previously unknown druggable Zn-sites in *multiple conserved* SARS-CoV-2 domains have been identified.

## Graphical abstract

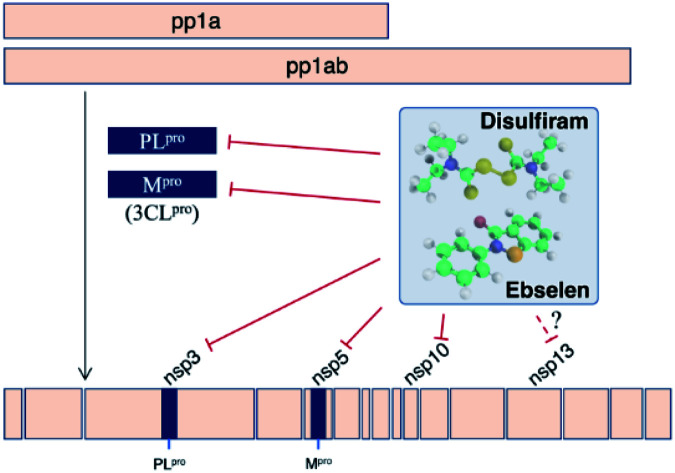

New infectious viruses causing epidemics/pandemics such as the SARS-CoV-2 require near-term effective and practical strategies to treat virus-infected patients. This is because developing specific antiviral drugs/vaccines takes time and in the interim, lives are lost/disrupted. One short-term strategy is to leverage the non-specificity of some FDA-approved drugs to target critical viral proteins.^1^ Here, we present a multi-targeting strategy combining evolutionary (conserved protein domains) and physical (factors controlling Zn^2+^-bound Cys reactivity) principles to identify new drug targets in *conserved* viral domains and applied it to the SARS-CoV-2. We show that clinically safe Zn-ejector drugs, disulfiram and ebselen, can target highly conserved Zn^2+^-binding and/or catalytic cysteines (Fig. 1) in *multiple conserved* viral domains essential for SARS-CoV-2 replication.

**Fig. 1:**
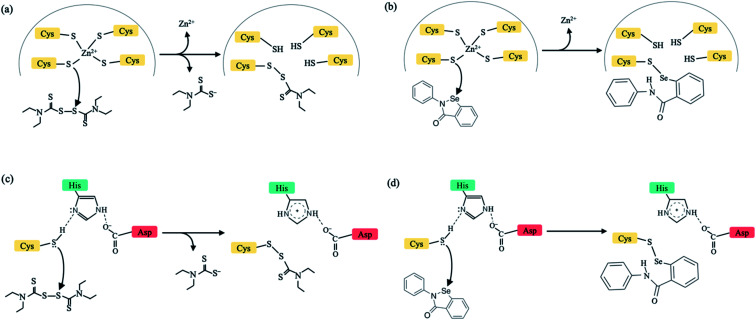
Schematic diagram to illustrate the mechanism of action by disulfiram (left) and ebselen (right) to release Zn^2+^ (a and b) or form a covalent adduct with a catalytic cysteine (c and d). In (a) and (c), half of disulfiram, diethyl-dithiol-carbamate, is covalently bonded to a Zn^2+^-bound/catalytic Cys.

Conserved cysteines and Zn^2+^ play crucial roles in viral infections.^2^ Cysteines can serve catalytic and/or structural roles (by binding metal ions and forming disulfide bridges) in viral enzymes/proteins. Zn^2+^ is an essential cofactor of many viral proteins, as it induces protein folding and stabilizes the local structure.^3,4^ Viral Zn-sites containing *reactive* cysteines bound to structural Zn^2+^ cations (*termed labile* Zn-sites) may serve as drug targets, as the Zn^2+^-bound thiolates can react with Zn-ejectors, leading to loss of Zn^2+^ and viral protein structure/function.^5–8^ Our previous studies^7,9^ had revealed the key factors determining which Zn^2+^-bound cysteines in proteins are reactive: labile Zn-sites are likely to be Zn-Cys_4_ or Zn-Cys_3_His sites lacking hydrogen bonds to any Zn-bound thiolate, which would reduce the Zn-bound thiolate's negative charge and reactivity (ESI Fig. S1†). We then used these guidelines to identify drug-target proteins containing *labile* structural Zn-sites.^8,10^

To circumvent toxicity due to undesirable targeting of essential human proteins, we had proposed using clinically safe (FDA-approved or in phase II/III clinical trials) Zn-ejecting agents that do not affect crucial host proteins (ESI Table S1†) to target putative labile Zn-sites in viral proteins.^8^ We showed that disulfiram, an anti-alcoholism drug, can eject Zn^2+^ from the predicted labile Zn-Cys_4_ site in the hepatitis C virus NS5A protein, inhibiting viral replication, and that inhibition was enhanced when disulfiram was combined with interferon-α.^8^ Following our work, disulfiram was found to eject Zn^2+^ and inhibit replication in other viruses, notably SARS- and MERS-CoV papain-like proteases (PL^pro^),^11^ which along with the main protease (M^pro^) cleave the pp1a and pp1ab replicase polyproteins into 16 nonstructural proteins (nsps).^12^ Since SARS-CoV PL^pro^ remained inactivated after removing unbound disulfiram, but was reactivated by reductant, disulfiram may also form a covalent adduct with the *catalytic* cysteine.^11^

The above findings suggest that a possible strategy to combat infectious coronaviruses with no approved drugs/vaccines is to employ clinically safe Zn-ejector drugs to target multiple essential Zn^2+^-bound and/or catalytic cysteines in conserved viral domains. Coronaviruses belonging to the order Nidovirales are amenable to our proposed strategy since they employ cysteine proteases (M^pro^, PL^pro^) and share conserved core replicase domains.^12^ Thus, we analyzed the SARS-CoV-2 genome (GenBank: MN908947.3) comprising 5′-methylated cap, genes encoding nonstructural and structural proteins, and 3′-polyadenylated tail. We focused on the large replicase polyprotein pp1ab because its cleavage products (nsp7–nsp16) are involved in viral replication.^12^ By searching the pp1ab sequence for conserved domains using the Conserved Domain Database,^13^ we found 18 such domains (ESI Table S2†). For each conserved domain found, we searched the Protein Data Bank (PDB)^14^ for <3 Å structures from other coronaviruses sharing similar function and high sequence identity using BLASTp^15^ (ESI Scheme S1†). We then checked each structure for Zn-(Cys_4_/Cys_3_His) sites, lacking hydrogen bonds to the Zn-bound thiolates. Although nsp12 (6nur, 6nus) and nsp14 (5c8s, 5c8t, 5c8u) structures have Zn-(Cys_4_/Cys_3_His) sites, their poor (≥3.1 Å) resolution prohibited reliable hydrogen-bond analysis of these Zn-sites.

Putative labile Zn-sites were found in the SARS-CoV structures of PL^pro^ subdomain of nsp3, nsp10 Zn-finger protein, and nsp13 helicase (Table 1). To obtain the corresponding SARS-CoV-2 sequences, we aligned the SARS-CoV PL^pro^/nsp10/nsp13 and the SARS-CoV-2 pp1ab sequences using BLASTp^15^ and obtained excellent alignment (ESI Table S3†). The SARS-CoV-2 PL^pro^ structure was homology-modeled from the SARS-CoV 4m0w_A structure using MODELLER,^16^ whereas the SARS-CoV-2 nsp10 and nsp13 structures were derived from the respective SARS-CoV structures (2xyq_B and 6jyt_B) by point mutations using SCRWL4 (ref. 17) since their sequences differ by only 1–2 residues. These modeled structures confirm the absence of hydrogen bonds to the Zn-bound thiolates (ESI Fig. S2†). Furthermore, model structures of the SARS-CoV-2 PL^pro^/M^pro^ with the catalytic cysteine covalently modified by disulfiram/ebselen obtained using QM/MM energy minimization show that the active site can accommodate a covalent adduct, thus inhibiting enzyme activity (ESI Fig. S3†).

**Table 1:** Predicted SARS-CoV-2 druggable Zn-sites and templates for building 3d-models

SARS-CoV-2 domain^a^	SARS-CoV protein	PDB structures^b^	Zn-ligands^c^
PL^pro^ subdomain of nsp3	PL^pro^	**4m0w**_A, 3e9s_A, 5tl7_B	C189, C192, C224, C226
nsp10	nsp10	**2xyq_B**, 2fyg_A, 5c8u_A, 5nfy_O, 5nfy_P, 2ga6_F, 2ga6_R, 5c8t_C	C117, C120, C128, C130
nsp13	Helicase	**6jyt_B**	C50, C55, C72, H75

a Conserved domain found by Conserved Domain Database.^13^

b PDB entry_chain ID of the SARS-CoV protein; that in bold was used to model the respective SARS-CoV-2 protein structure.

c Residue numbers correspond to those of the respective SARS-CoV-2 protein.

Among the three predicted SARS-CoV-2 targets, we chose nsp10 and the PL^pro^ subdomain of nsp3 to validate our predictions. We subcloned the cDNAs of SARS-CoV-2 PL^pro^ and nsp10 (residues 1541–1855 and 4231–4362 of pp1a/pp1ab, respectively) and expressed them in *E. coli*. Each purified recombinant protein (5 μM) was incubated with ten Zn^2+^-ejecting compounds (5 μM), including disulfiram and ebselen, and Zn^2+^ release was monitored by the increase in fluorescence emission signal from the zinc-specific fluorophore, FluoZin-3 (1 μM). Among the ten Zn^2+^-ejectors tested, disulfiram, ebselen, 5,5′-dithiobis(2-nitrobenzoic acid) (DTNB), 2,2′-dithiodipyridine, and 2,2′-dithiobis(benzothiazole) could eject Zn^2+^ from PL^pro^ (Fig. 2a) and nsp10 (Fig. 2b).

**Fig. 2:**
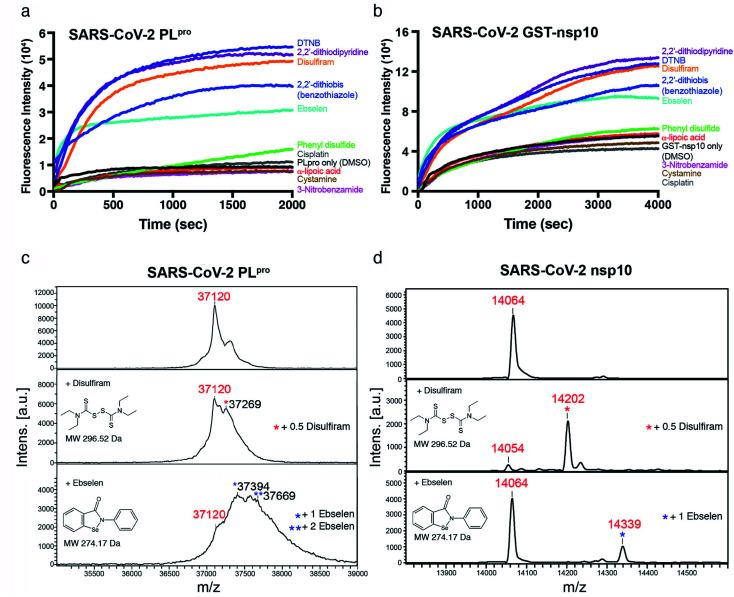
Zn^2+^ release from SARS-CoV-2 PL^pro^ and nsp10 by Zn^2+^-ejecting compounds. Upon adding each Zn^2+^-ejecting agent (5 μM) to PL^pro^ (5 μM) (a), or GST-fused nsp10 (5 μM) (b), Zn^2+^ release was detected by the increase of the fluorescence signal from FluoZin-3 (1 μM) with emission and excitation wavelengths of 494 nm and 516 nm, respectively. The MWs of PL^pro^ (c) and nsp10 (d) before (top) and after adding disulfiram (middle) or ebselen (bottom) were measured by MALTI-TOF mass spectrometry. Three independent experiments gave similar results hence only spectra from one of the experiments are shown.

To confirm that disulfiram and ebselen are covalently bound to the cysteines in PL^pro^ and nsp10, the molecular weights (MWs) of PL^pro^ and nsp10 before and after adding these two zinc-ejectors were measured by mass spectrometry. The PL^pro^ MALTI-TOF mass spectrum (Fig. 2c, top panel) revealed a major peak with a measured MW of 37 120 Da close to PL^pro^'s calculated MW (37 125 Da). Disulfiram-treated PL^pro^ (Fig. 2c, middle panel) had an additional peak at 37 269 Da, suggesting half of disulfiram was bound in PL^pro^. Ebselen-treated PL^pro^ (Fig. 2c, bottom panel) had additional peaks at 37 394 and 37 669 Da, indicating one and two ebselen molecules were bound, respectively. For nsp10 with a calculated MW of 14 066 Da, the MALTI-TOF mass spectra (Fig. 2d) exhibited additional peaks at 14 202 Da (corresponding to 0.5 disulfiram-bound nsp10) and 14 339 Da (corresponding to one ebselen-bound nsp10), suggesting that both drugs were covalently bound to cysteines in nsp10. To further verify that the drug was attached to cysteines involved in Zn^2+^-binding in PL^pro^, disulfiram- and ebselen-treated PL^pro^ were digested by trypsin, and analyzed by liquid chromatography-tandem mass spectrometry (LC-MS/MS). The MWs of the PL^pro^ peptides, ^183^RVLNVV**C**K^190^ (1076.6 Da) and ^191^T**C**GQQQTTLK^200^ (1253.6 Da) with an additional MW of 148 Da, confirm that the Zn^2+^-bound C189 and C192 were covalently linked to half of disulfiram. Likewise, the MWs of the PL^pro^ peptides, ^184^VLNVV**C**K^190^ (1048.4 Da) and ^191^T**C**GQQQTTLK^200^ (1381.5 Da) with an additional MW of 274 Da, show that the Zn^2+^-bound C189 and C192 were bonded to ebselen. These results confirm that disulfiram and ebselen can covalently bind Zn^2+^-bound cysteines in SARS-CoV-2 PL^pro^, resulting in the loss of Zn^2+^.

Since disulfiram and ebselen are in clinical use, we further determined if they could inhibit PL^pro^ by measuring protease activity in cleaving a fluorogenic substrate (Dabcy-FTLKGG↓APTKVTE-Edans) in the absence and presence of varying concentrations of each Zn^2+^-ejector drug. We found that disulfiram and ebselen inhibited PL^pro^ activity with IC_50_ of 7.52 ± 2.13 μM and 2.36 ± 0.16 μM, respectively (Fig. 3a and b). Interestingly, compared to disulfiram, ebselen displayed slightly stronger inhibition of PL^pro^ activity despite its less potent Zn-ejecting ability (Fig. 2a). In SARS-CoV-2 M^pro^ that lacks a Zn^2+^-site, ebselen (IC_50_ = 0.67 ± 0.09 μM) also showed stronger inhibition than disulfiram (IC_50_ = 9.35 ± 0.18 μM).^18^ This suggests that ebselen may be more effective in targeting the catalytic cysteine than disulfiram.

**Fig. 3:**
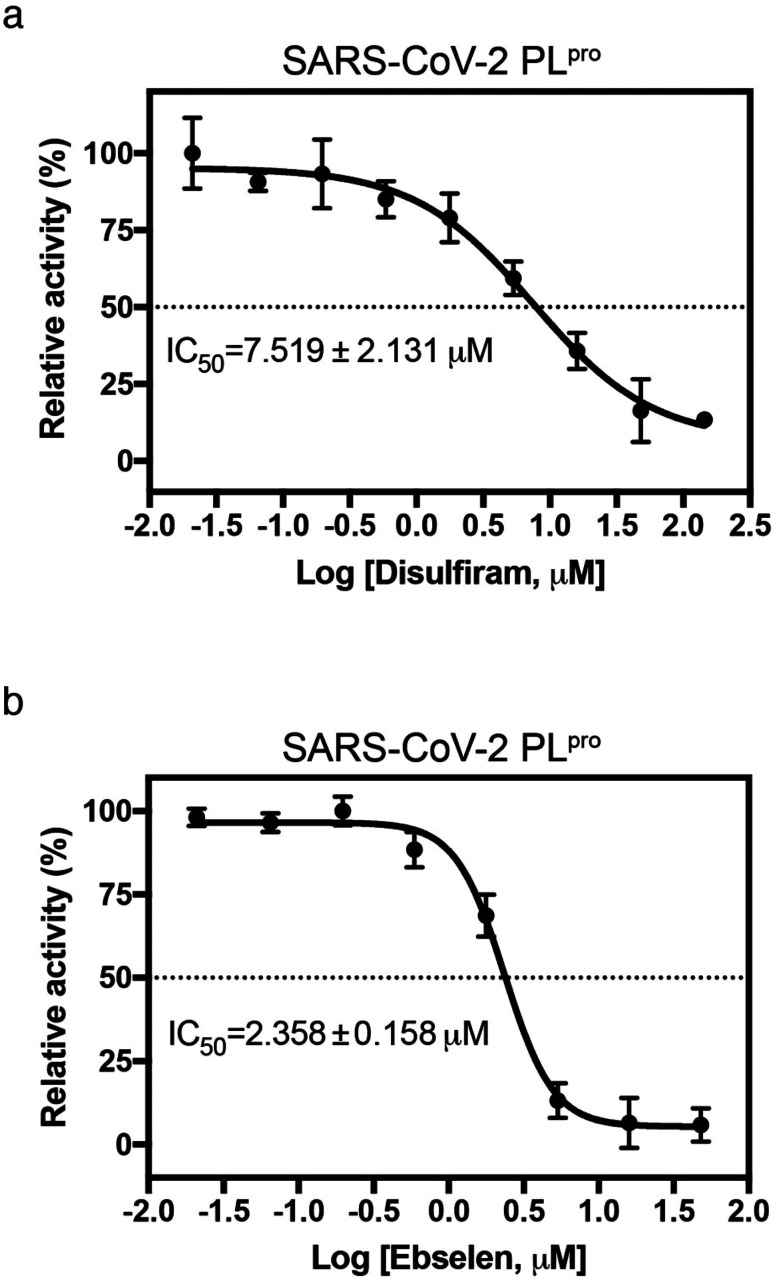
Inhibition of SARS-CoV-2 PL^pro^ by disulfiram and ebselen. The protease activity of PL^pro^ (0.5 μM) in the presence of 0–144 μM disulfiram (a) or 0–48 μM ebselen (b) was assayed using a fluorogenic substrate (50 μM, Dabcy-FTLKGGAPTKVTE-Edans-NH2). The IC_50_ value was determined by sigmoidal nonlinear regression logistic four parameter analyses using GraphPad Prim software (*n* = 3, error bars denote standard deviation).

To our knowledge, we are the first to combine knowledge of conserved coronavirus domains and the key factors controlling Zn-bound cysteine reactivity^9^ to identify previously unknown druggable Zn-sites in multiple SARS-CoV-2 domains. The labile Zn-sites discovered in SARS-CoV-2 are attractive drug targets, as they are highly conserved among coronaviruses and play vital structural/catalytic roles in key proteins in the viral life cycle: the Zn-binding ability of PL^pro^ is crucial for structural integrity and protease activity.^19^ PL^pro^ not only cleaves the viral polyproteins, but also helps SARS/MERS-CoV to evade the host innate immune response through deubiquitinating and deISGylating enzymatic activities.^19,20^ The labile Zn-site in nsp10, a crucial cofactor for multiple replicative enzymes such as nsp14 and nsp16,^21^ plays an important structural role.^22^ The Zn-binding domain of nsp13 helicase, which catalyzes dsRNA/dsDNA unwinding, is vital for helicase activity.^23^

Whereas most studies focus on designing inhibitors or repurposing drugs to target a specific viral enzyme/protein such as M^pro^,^1^ our study shows that the same Zn-ejecting drug can attack highly conserved cysteines in *multiple* viral targets. Disulfiram, used since 1951 with a recommended daily dose of ≤500 mg, and ebselen are considered to be clinically safe.^24,25^ Both may serve as multi-targeting drugs acting at various stages of the virus life cycle: they can target Zn-bound cysteines in PL^pro^, nsp10, and possibly nsp13, and/or catalytic cysteines in PL^pro^ and M^pro^ enzyme. Crippling both PL^pro^ and M^pro^ enzymes needed to cleave the replicase polyprotein 1a and pp1ab would likely inhibit SARS-CoV-2 replication. Indeed, both disulfiram and ebselen were found to decrease the number of viral RNA copies at 10 μM concentration in SARS-CoV-2-infected Vero E6 cells and ebselen was further shown to inhibit SARS-CoV-2 with a EC_50_ of 4.67 ± 0.80 μM in plaque-reduction assay.^18^ Disulfiram/ebselen may serve as a broad-spectrum anti-viral since the domains containing the labile Zn-sites are highly *conserved* across several types of coronaviruses: disulfiram can inhibit SARS-CoV, MERS-CoV, and SARS-CoV-2 PL^pro^*in vitro*, whereas most SARS-CoV PL^pro^ inhibitors are inactive against MERS-CoV PL^pro^.^26^

A possible advantage of targeting *multiple conserved* domains is that the virus has to undergo simultaneous appropriate mutations of the different targeted domains to develop drug resistance.^8^ We propose combining disulfiram/ebselen with other FDA-approved drugs, which have immune-modulatory/anti-inflammatory properties and/or anti-viral effect, to potentially inhibit SARS-CoV-2 replication synergistically. To test this possibility, we chose the zinc ionophore, hydroxychloroquine, because it can downregulate pro-inflammatory cytokines^27^ and can increase the Zn^2+^ level inside a cell.^28^ Increasing intracellular Zn^2+^ concentration has been shown to inhibit SARS-CoV nsp12 RNA-dependent RNA polymerase, the core enzyme of a multiprotein replication and transcription complex.^29^ We evaluated antiviral synergy between disulfiram/ebselen and hydroxychloroquine by pretreating Vero E6 cells with the two drugs at various concentrations for 1 h at 37 °C, followed by incubation with SARS-CoV-2 for 1 day at 37 °C (see Methods). We then determined the anti-SARS-CoV-2 activity of each drug or drug combination using immunofluorescence assay to detect SARS-CoV-2 N protein expression (green in Fig. 4a). Consistent with previous results,^18^ disulfiram and ebselen exhibit an estimated IC_50_ of 17.5 μM and 23.3 μM, respectively, based on the quantification of SARS-CoV-2 N protein expression (Fig. 4b). The SARS-CoV-2 infection rate in Vero E6 cells treated with a given concentration of hydroxychloroquine and/or disulfiram/ebselen is shown as the mean and corresponding standard deviation of three replicates in Fig. 4c. Disulfiram/ebselen combined with hydroxychloroquine exhibited enhanced antiviral effect compared to each drug alone with *p* values < 0.05. For example, 12.5 μM disulfiram combined with 5 μM hydroxychloroquine did not affect cell viability, but reduced viral infection compared to disulfiram alone (*p* value = 0.007) or hydroxychloroquine alone (*p* value = 0.014). This provides proof-of-concept for combining disulfiram/ebselen with other safe drugs to synergistically inhibit SARS-CoV-2 by targeting multiple conserved viral regions/pathways.

**Fig. 4:**
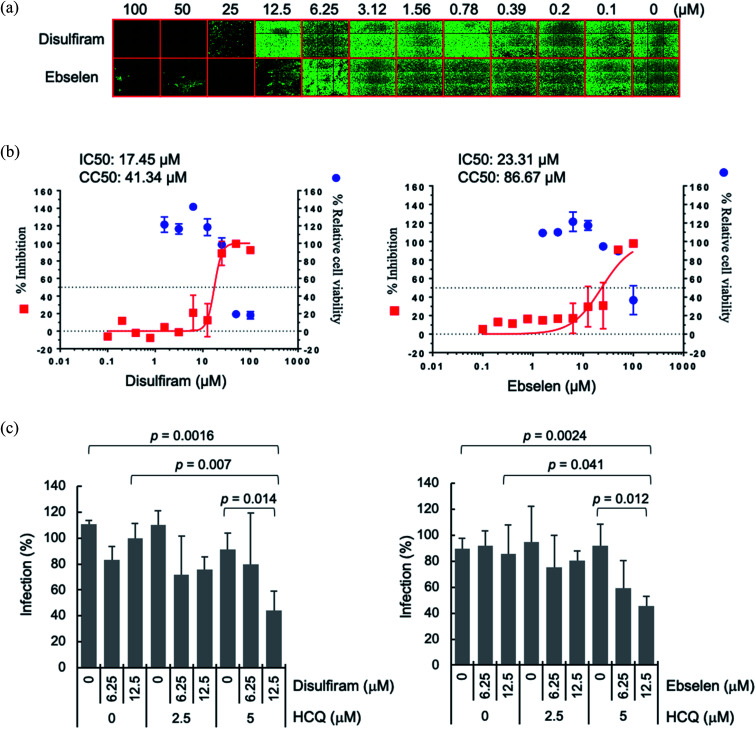
Synergistic antiviral potential of disulfiram/ebselen and hydroxychloroquine (HCQ). (a) The antiviral activities of disulfiram and ebselen against SARS-CoV-2 were determined on Vero E6 cells using immunofluorescence assay to detect SARS-CoV-2 N protein expression (green). (b) Viral infection was quantified by a high-content image analysis system and the average infection rate of no drug treatment was set as 100% for calculation of the 50% inhibitory concentration (IC_50_). For the 50% cytotoxic concentration (CC_50_), Vero E6 cells treated with the indicated compound were assayed by Cell Counting Kit-8. IC_50_ and CC_50_ were calculated by Prism software. (c) The SARS-CoV-2 infection rates in Vero E6 cells treated with hydroxychloroquine (HCQ) plus disulfiram or ebselen were determined as described above, and shown as means and standard deviations (*n* = 3). The average infection rate of six sets of experiments with no drug treatment was set as 100%. The *p* values were calculated by student's *t* test.

In summary, this study offers a possible strategy to tackle outbreaks of coronaviruses by leveraging the non-specificity of clinically safe Zn-ejector drugs combined with broad-spectrum antivirals to target *multiple* conserved domains essential for viral replication. Our general strategy based on evolutionary and physical principles can be used to identify druggable Zn-sites in other non-coronaviruses employing essential cysteines and Zn^2+^ in conserved viral domains.

## Conflicts of interest

The authors declare no competing financial interests.

## Supplementary Material

SC-011-D0SC02646H-s001
