# Correction: Multi-targeting of functional cysteines in multiple conserved SARS-CoV-2 domains by clinically safe Zn-ejectors

**DOI:** 10.1039/d1sc90081a

**Published:** 2021-04-22

**Authors:** Karen Sargsyan, Chien-Chu Lin, Ting Chen, Cédric Grauffel, Yi-Ping Chen, Wei-Zen Yang, Jian-Jong Liang, Chun-Che Liao, Yi-Ling Lin, Hanna S. Yuan, Carmay Lim

**Affiliations:** Institute of Biomedical Sciences, Academia Sinica Taipei 115 Taiwan carmay@gate.sinica.edu.tw; Institute of Molecular Biology, Academia Sinica Taipei 115 Taiwan hanna@sinica.edu.tw; Department of Chemistry, National Tsing Hua University Hsinchu 300 Taiwan

## Abstract

Correction for ‘Multi-targeting of functional cysteines in multiple conserved SARS-CoV-2 domains by clinically safe Zn-ejectors’ by Karen Sargsyan *et al.*, *Chem. Sci.*, 2020, **11**, 9904–9909, DOI: 10.1039/D0SC02646H.

The authors regret the omission of three of the authors, Jian-Jong Liang, Chen-Che Liao and Yi-Lang Lin, from the original manuscript. The corrected list of authors and affiliations for this paper is as shown above.

The Royal Society of Chemistry apologises for these errors and any consequent inconvenience to authors and readers.

