# Supplementary material for: Multi-targeting of functional cysteines in multiple conserved SARS-CoV-2 domains by clinically safe Zn-ejectors
Source: Chem Sci. 2020 Sep 1;11(36):9904–9. doi: 10.1039/d0sc02646h (PMC8162115; doi:10.1039/d0sc02646h)
Supplement: SC-011-D0SC02646H-s001 [file SC-011-D0SC02646H-s001.pdf]

Electronic Supplementary Material (ESI) for Chemical Science.
This journal is © The Royal Society of Chemistry 2020

Supporting Information

Multi-Targeting of Functional Cysteines in Multiple Conserved SARS-CoV-2 Domains by Clinically Safe Zn-ejectors

Karen Sargsyan[1#], Chien-Chu Lin[2#], Ting Chen[1#], Cédric Grauffel[1], Yi-Ping Chen[2], Wei-Zen Yang[2], Hanna S. Yuan[2*], and Carmay Lim[1,3*]

[1]Institute of Biomedical Sciences, Academia Sinica, Taipei 115, Taiwan

[2]Institute of Molecular Biology, Academia Sinica, Taipei 115, Taiwan

[3]Department of Chemistry, National Tsing Hua University, Hsinchu 300 Taiwan

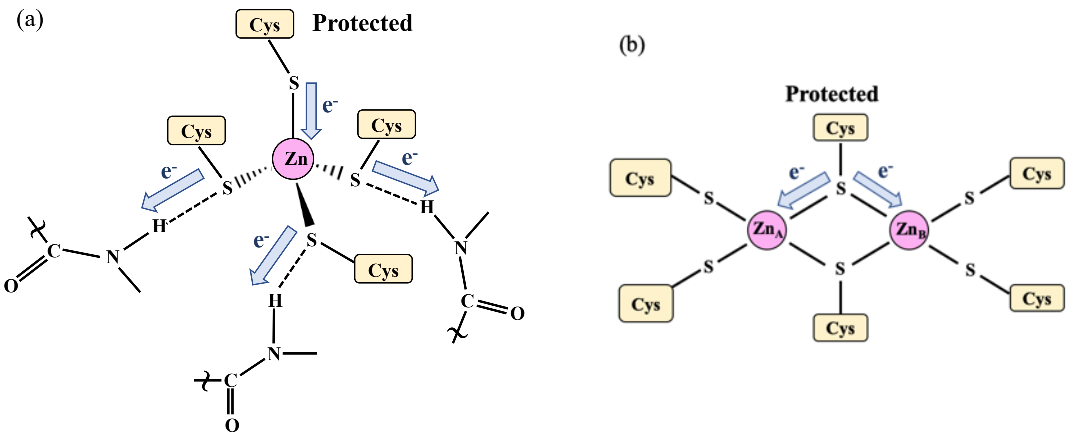

Supplementary Figure S1. Hydrogen bonds to Zn-bound Cys(S^-) (a) or a second Zn^{2+} (b) reduce the negative charge of all Zn-bound Cys(S^-), including those with no hydrogen-bonding interactions, suppressing their reactivity. Arrows indicate the charge flow from Cys(S^-) to the more electronegative O.

Supplementary Table S1. Zn-ejectors that are FDA-approved or in clinical trials

Drug name (other name)	Chemical structure	Indication(s)	Drug Status	Reference for Zn-ejection
Disulfiram (Antabuse) (Antabus)	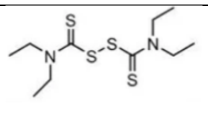	Chronic alcoholism	Approved in 1951	McDonnell *et al.* [1]
Cisplatin (CDDP)	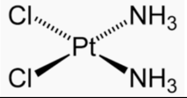	Anticancer	Approved in 1978	Lenstra *et al.* [2]
Iniparib (BSI-201)	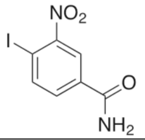	Anticancer	Phase III[a]	Mateo *et al.* [3]
Ebselen (PZ-51) (SPI-1005)	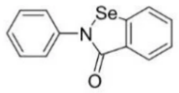	Meniere's disease; hearing loss; Bipolar disorder	Phase II Completed	Sekirnik *et al.* [4]

[a]Iniparib failed Phase III trials because it is a poor inhibitor of PARP activity, not safety issues.

Supplementary Table S2. Conserved domains found in pp1ab polyprotein using best scoring domain model.

Conserved domain name	Accession	Description or notes	Interval*	E-value
nsp1 super family	cl13018	Nonstructural protein nsp1	13–127	$3.67e^{-63}$
DUF3655 super family	cl13772	Protein of unknown function (DUF3655)	920–987	$1.44e^{-19}$
Macro	pfam01661	Macro domain	1058–1165	$2.31e^{-22}$
SUD-M super family	cl13138	Single-stranded poly(A) binding domain	1351–1493	$2.73e^{-75}$
nsp3_PL2pro super family	cl13549	Coronavirus polyprotein cleavage domain of nsp3	1498–1561	$1.42e^{-27}$
Viral_protease super family	cl09459	Papain like viral protease	1564–1880	$3.80e^{-132}$
NAR super family	cl24732	Nucleic acid-binding domain (NAR)	1907–2019	$2.26e^{-12}$
Corona_nsp4_C	pfam16348	Coronavirus nonstructural protein 4 C-terminus	3169–3261	$3.84e^{-43}$
nsp5 Peptidase_C30	pfam05409	Coronavirus endopeptidase C30, also called main (M^{pro}) or 3C-like protease ($3CL^{pro}$)	3292–3569	$7.31e^{-162}$
nsp7	pfam08716	nsp7 replicase	3860–3942	$1.29e^{-38}$
nsp8	pfam08717	nsp8 replicase	3943–4140	$1.83e^{-113}$
nsp9	pfam08710	nsp9 replicase	4141–4253	$6.77e^{-55}$
nsp10	pfam09401	RNA synthesis protein nsp10	4265–4384	$1.01e^{-71}$
nsp12 Corona_RPol_N	pfam06478	Coronavirus RPol N-terminus	4406–4758	0
nsp13_DEXXQc_Upf1-	cd17934	DEXXQ-box	5596–5767	$9.68e^{-20}$

like		helicase domain of Upf1-like helicase		
nsp13_DNA2	COG1112	Superfamily I DNA and/or RNA helicase	5647–5916	$3.79e^{-13}$
nsp11	pfam06471	This region encodes the nsp14 protein	5928–6520	0
nsp13	pfam06460	This region encodes the nsp16 protein	6800–7095	0

*Interval – starting and ending position of the domain in the polyprotein1ab sequence.

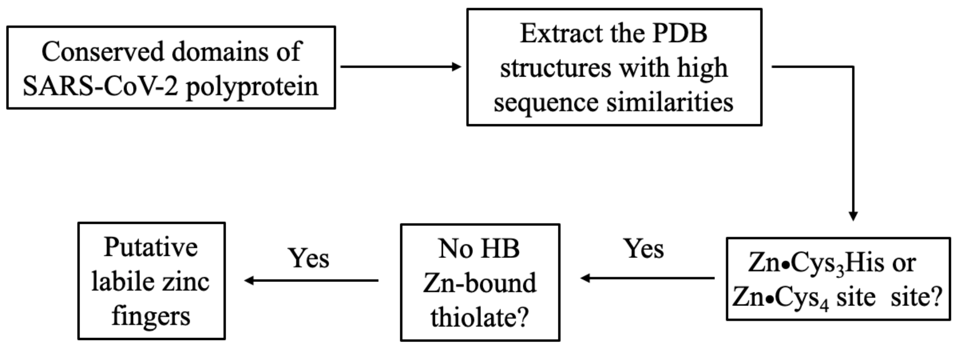

Supplementary Scheme S1. Flowchart for searching PDB structures with putative labile Zn-sites. HBPLus[5] was used to compute hydrogen bonds to the Zn-bound thiolate.

Supplementary Table S3. Sequence alignments between SARS-CoV-2 (denoted as 2019-nCoV) and SARS-CoV, (a) papain-like protease, (b) nsp10 and (c) nsp13. The putative labile Zn-bound ligands are shaded in grey.

(a)

```
2019-nCoV  EVRTIKVFTTVDNINLHTQVVDMSMTYGQQFGPTYLDGADVTKIKPHNSHEGKTFYVLPNDDTLRVEAFEYYHTTDPSFL
SARS-CoV   EVKTIKVFTTVDNTNLHTQLVDMSMTYGQQFGPTYLDGADVTKIKPHVNHEGKTFFVLPSDDTLRSEAFEYYHTLDESFL
           **:********** *****:*************************** .******:***.***** ******** * ***

2019-nCoV  GRYMSALNHTKKWKYPQVNGLTSIKWADNNCYLATALLTLQQIELKFNPPALQDAYYRARAGEAANFCALILAYCNKTVG
SARS-CoV   GRYMSALNHTKKWKFPQVGGLTSIKWADNNSYLSSVLLALQQLEVKFNAPALQEAYYRARAGDAANFCALILAYSNKTVG
           **************:***.***********.**::.**:***:*:***.****:********:***********.*****

2019-nCoV  ELGDVRETMSYLFQHANLDSCKRVLNVVCKTCGQQQTTLKGVEAVMYMGTLSYEQFKKGVQIPCTCGKQATKYLVQQESP
SARS-CoV   ELGDVRETMTHLLQHANLESAKRVLNVVCKHCGQKTTTLTGVEAVMYMGTLSYDNLKTGVSIPCVCGRDATQYLVQQESS
           *********::*:*****:*.********* ***: ***.*************:::*.**.***.**::**:*******.

2019-nCoV  FVMMSAPPAQYELKHGTFTCASEYTGNYQCGHYKHITSKETLYCIDGALLTKSSEYKGPITDVFYKENSYTTTIKPVT
SARS-CoV   FVMMSAPPAEYKLQQGTFLCANEYTGNYQCGHYTHITAKETLYRIDGAHLTKMSEYKGPVTDVFYKETSYTTTIKPVS
           *********:*:*::*** **.***********.***:***** **** *** ******:*******.*********:
```

(b)

```
2019-nCoV  TVLSFCAFAVDAAKAYKDYLASGGQPITNCVKMLCTHTGTGQAITVTPEANMDQESFGGASCCLYCRCHIDHPNPKGF
SARS-CoV   TVLSFCAFAVDPAKAYKDYLASGGQPITNCVKMLCTHTGTGQAITVTPEANMDQESFGGASCCLYCRCHIDHPNPKGF
           ***********.******************************************************************

2019-nCoV  CDLKGKYVQIPTTCANDPVGFTLKNTVCTVCGMWKGYGCSCD
SARS-CoV   CDLKGKYVQIPTTCANDPVGFTLRNTVCTVCGMWKGYGCSCD
           ***********************:******************
```

(c)

```
2019-nCoV  NSAVGACVLCNSQTSLRCGACIRRPFLCCKCCYDHVISTSHKLVLSVNPYVCNAPGCDVTDVTQLYLGGMSYYCKSHKPP
SARS-CoV   NSAVGACVLCNSQTSLRCGACIRRPFLCCKCCYDHVISTSHKLVLSVNPYVCNAPGCDVTDVTQLYLGGMSYYCKSHKPP
           ********************************************************************************

2019-nCoV  ISFPLCANGQVFGLYKNTCVGSDNVTDFNAIATCDWTNAGDYILANTCTERLKLFAAETLKATEETFKLSYGIATVREVL
SARS-CoV   ISFPLCANGQVFGLYKNTCVGSDNVTDFNAIATCDWTNAGDYILANTCTERLKLFAAETLKATEETFKLSYGIATVREVL
           ********************************************************************************

2019-nCoV  SDRELHLSWEVGKPRPPLNRNYVFTGYRVTKNSKVQIGEYTFEKGDYGDAVVYRGTTTYKLNVGDYFVLTSHTVMPLSAP
SARS-CoV   SDRELHLSWEVGKPRPPLNRNYVFTGYRVTKNSKVQIGEYTFEKGDYGDAVVYRGTTTYKLNVGDYFVLTSHTVMPLSAP
           ********************************************************************************

2019-nCoV  TLVPQEHYVRITGLYPTLNISDEFSSNVANYQKVGMQKYSTLQGPPGTGKSHFAIGLALYYPSARIVYTACSHAAVDALC
SARS-CoV   TLVPQEHYVRITGLYPTLNISDEFSSNVANYQKVGMQKYSTLQGPPGTGKSHFAIGLALYYPSARIVYTACSHAAVDALC
           ********************************************************************************

2019-nCoV  EKALKYLPIDKCSRIIPARARVECFDKFKVNSTLEQYVFCTVNALPETTADIVVFDEISMATNYDLSVVNARLRAKHYVY
SARS-CoV   EKALKYLPIDKCSRIIPARARVECFDKFKVNSTLEQYVFCTVNALPETTADIVVFDEISMATNYDLSVVNARLRAKHYVY
           ********************************************************************************

2019-nCoV  IGDPAQLPAPRTLLTKGTLEPEYFNSVCRLMKTIGPDMFLGTCRRCPAEIVDTVSALVYDNKLKAHKDKSAQCFKMFYKG
SARS-CoV   IGDPAQLPAPRTLLTKGTLEPEYFNSVCRLMKTIGPDMFLGTCRRCPAEIVDTVSALVYDNKLKAHKDKSAQCFKMFYKG
           ********************************************************************************

2019-nCoV  VITHDVSSAINRPQIGVVREFLTRNPAWRKAVFISPYNSQNAVASKILGLPTQTVDSSQGSEYDYVIFTQTTETAHSCNV
SARS-CoV   VITHDVSSAINRPQIGVVREFLTRNPAWRKAVFISPYNSQNAVASKILGLPTQTVDSSQGSEYDYVIFTQTTETAHSCNV
           ********************************************************************************

2019-nCoV  NRFNVAITRAKVGILCIMSDRDLYDKLQFTSLEIPRRNVATLQ
SARS-CoV   NRFNVAITRAKIGILCIMSDRDLYDKLQFTSLEIPRRNVATLQ
           ***********:*******************************
```

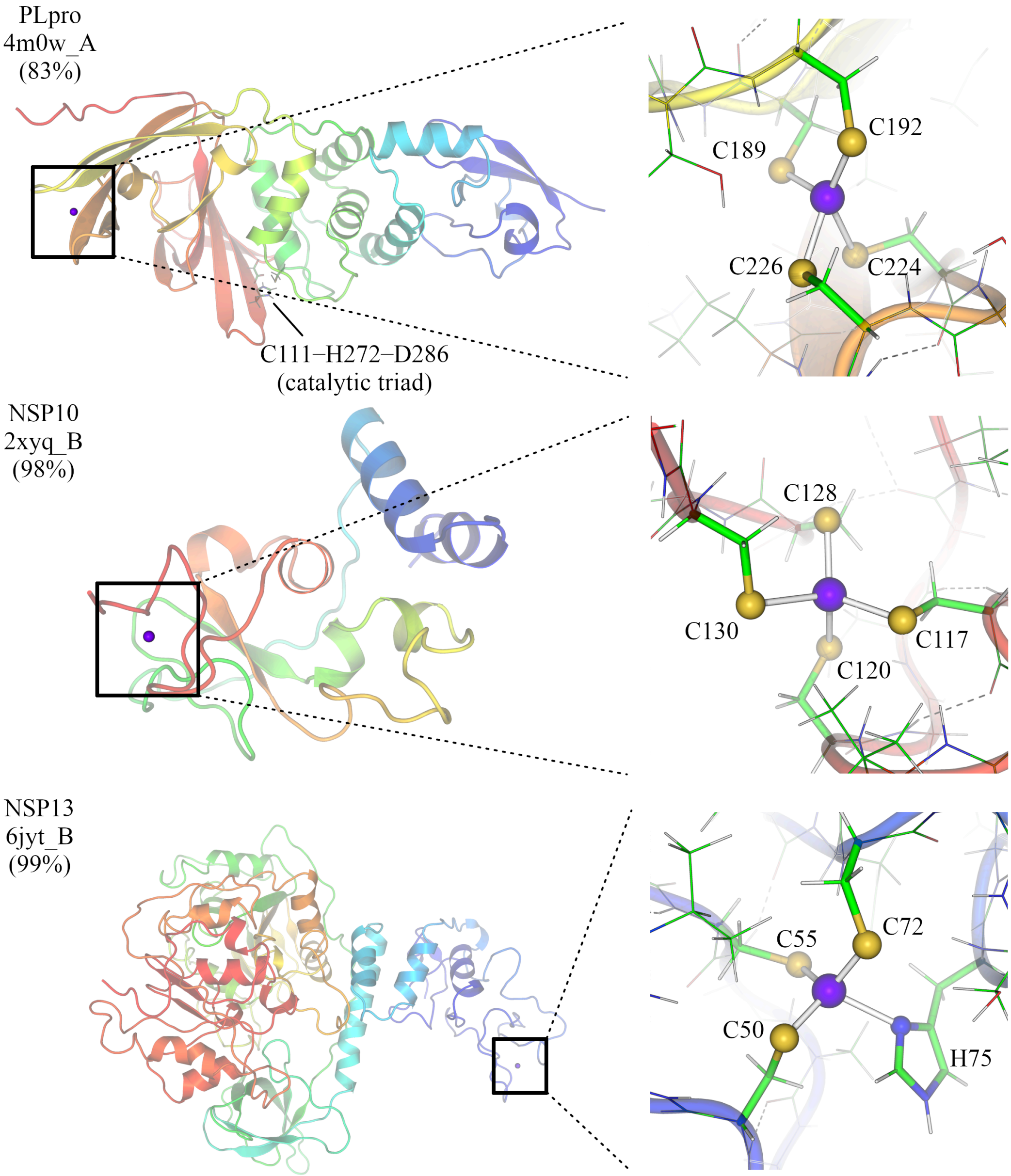

Supplementary Figure S2. Model 3d-structures of SARS-CoV-2 drug target proteins and their putative labile Zn-sites. The SARS-CoV-2 nsp13 and nsp10 structures were derived from the respective 6jyt_B and 2xyq_B SARS-CoV structures by point mutations using SCRWL4,[6] as their sequences differ from the corresponding SARS-CoV sequences by one and two residues whose C^{α} atoms are > 16 and 75 Å from Zn^{2+}, respectively. The structure of SARS-CoV-2 papain-like protease (PLpro) was derived from that of the SARS-CoV PLpro (4m0w_A) using MODELLER.[7] The % numbers in parentheses is % sequence identity between the SARS-CoV-2 and template sequences. Each structure is depicted using a color gradient from blue (N-terminus) to red (C-terminus).

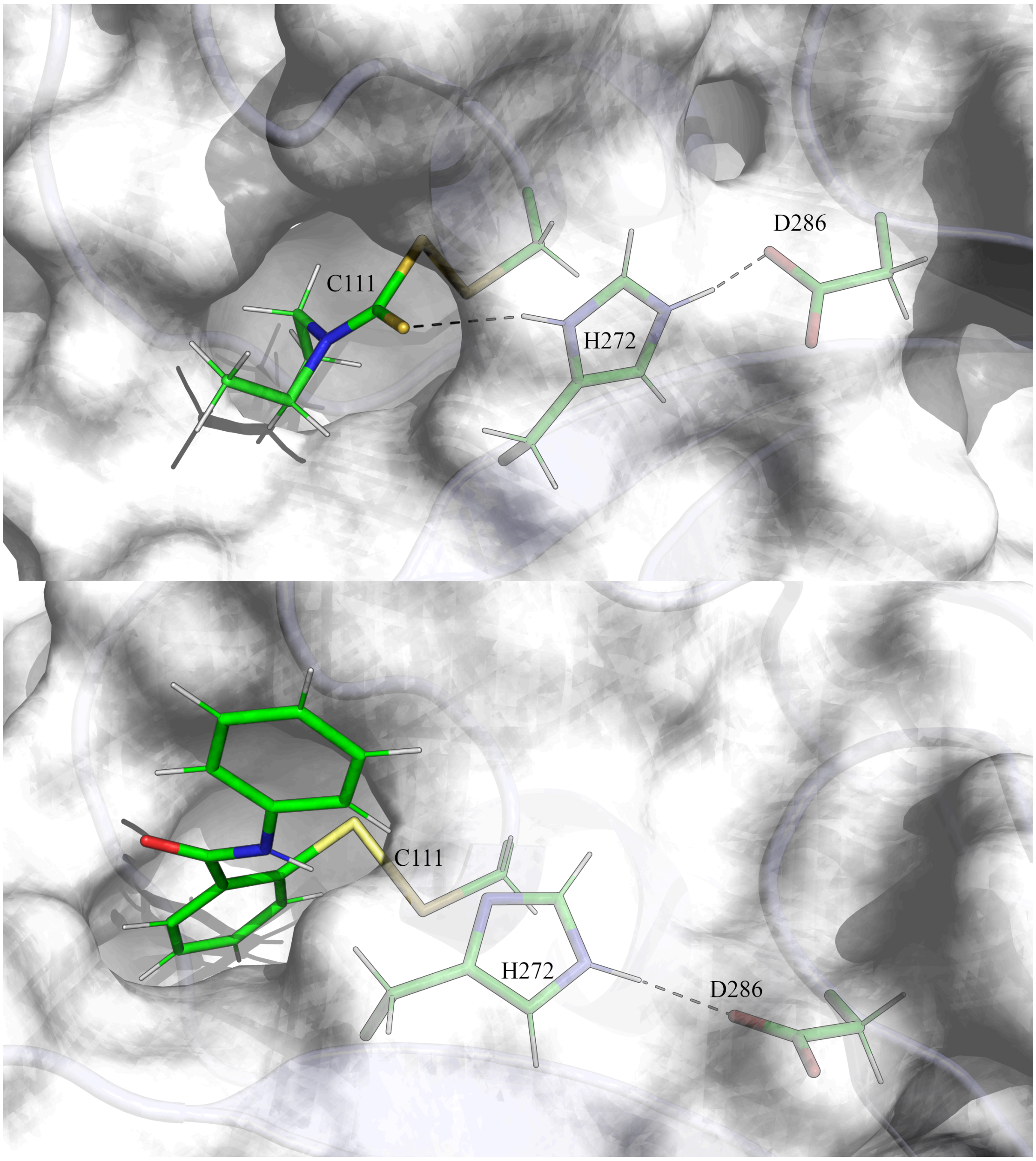

Supplementary Figure S3a. Model 3d-structure of the SARS-Cov-2 PL^{pro} active site after disulfiram (top) and ebselen (bottom) have formed a covalent bond with the catalytic cysteine, C111. The structure was obtained by two cycles of QM/MM energy minimization: First, the QM region, comprising residues C111, H272, N109 and W106, was optimized for a total of 500 steps, then the rest of the MM system was minimized by progressively reducing harmonic restraints on the backbone and side chains. This procedure was then repeated. Residues comprising the catalytic triad, C111, H272, and D286, are shown in ball and stick format with hydrogen in white, carbon in green, nitrogen in blue, oxygen in red, and sulphur in yellow.

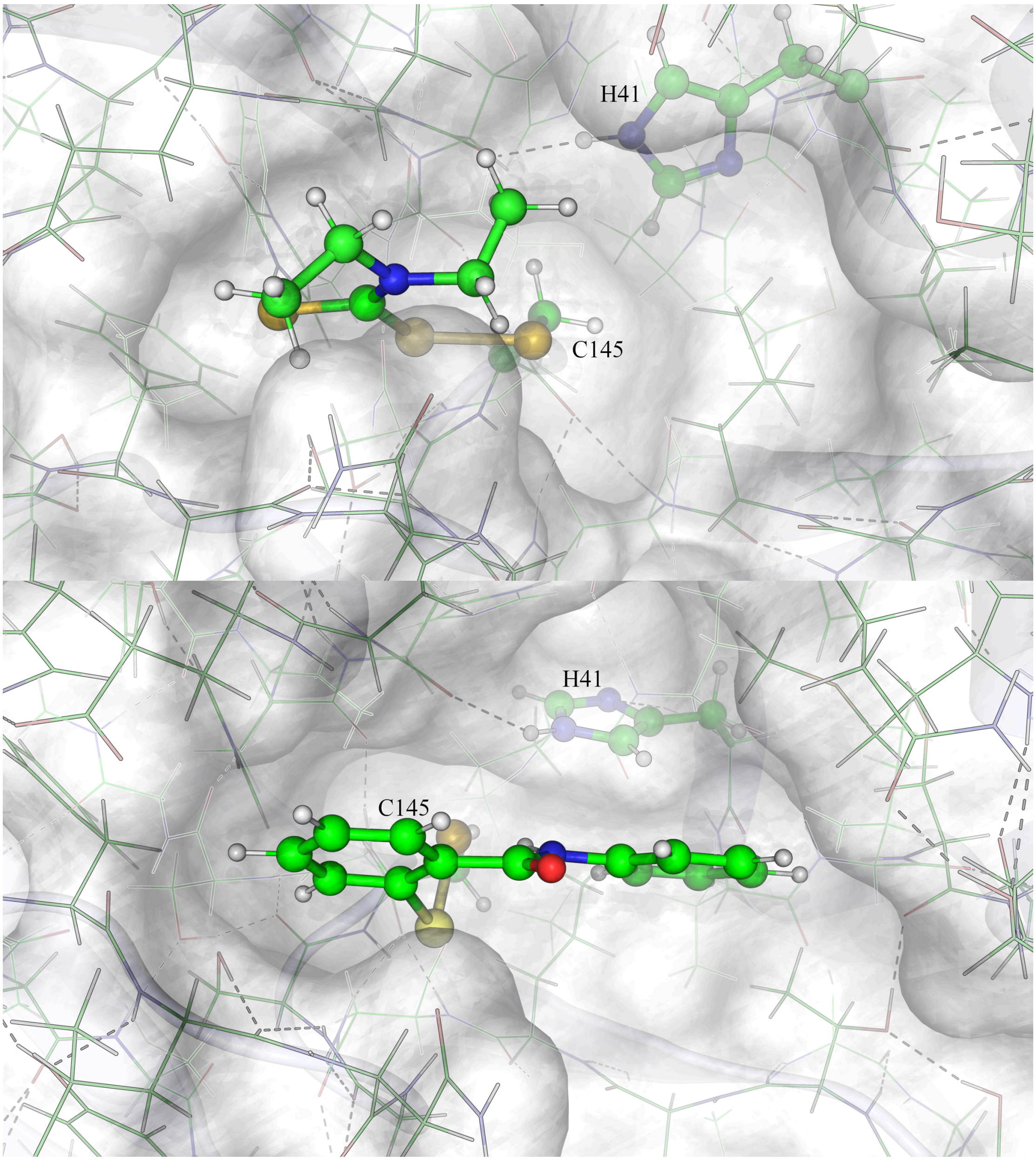

Supplementary Figure S3b. Model 3d-structure of the SARS-Cov-2 M^{pro} active site after disulfiram (top) and ebselen (bottom) have formed a covalent bond with the catalytic cysteine, C145. The structure was obtained by two cycles of QM/MM energy minimization: First, the QM region, comprising residues C145 and H163, was optimized for a total of 500 steps, then the rest of the MM system was minimized by progressively reducing harmonic restraints on the backbone and side chains. This procedure was then repeated. Residues comprising the catalytic dyad, C145 and H41 are shown in ball and stick format with hydrogen in white, carbon in green, nitrogen in blue, oxygen in red, and sulphur in yellow.

General Materials and Methods

Protein expression and purification. The cDNA of SARS-CoV-2 nsp3 papain-like cysteine protease (PL^{pro}) subdomain (residues 1541–1855 of pp1a/pp1ab) and nsp10 (residues 4231-4362 pp1a/pp1ab) were synthesized from MDBio, Inc. (Taiwan) and cloned respectively into BamHI/EcoRI sites of the expression vector pET22b and NdeI/XhoI sites of a modified expression vector pGEX-4T-1 that expresses an N-terminal GST tag followed by a TEV protease cleavage site. Both plasmids were transformed into *E. coli* BL21 (DE3) pLysS strain cultured in LB medium supplemented with 100 μg/ml ampicillin. Cells were grown to an optical density of 0.6 measured at a wavelength of 600 nm and induced by 0.8 mM IPTG at 18°C for 14−16 h to express the SARS-CoV-2 His-tagged nsp3 PL^{pro} domain and GST-fused nsp10.

The harvested cells were disrupted by a microfluidizer (Microfluidics M-110P) in the lysis buffer containing 1xPBS and 10 mM β-mercaptoethanol. For the purification of the His-tagged SARS-CoV-2 PL^{pro}, the cell lysate was centrifuged and the supernatant was loaded onto a HisTrap FF column (5 mL, GE HealthCare), and washed with a washing buffer containing 50 mM Tris-HCl (pH 8.0), 150 mM NaCl, 10 mM β-mercaptoethanol, and 40 mM imidazole. PL^{pro} protein sample was then eluted by a buffer of 50 mM Tris-HCl (pH 8.0), 150 mM NaCl, 10 mM β-mercaptoethanol, and 250 mM imidazole. The protein sample was collected and further loaded into a gel filtration column (HiLoad 16/60 Superdex 200, GE HealthCare) and eluted by a buffer of 50 mM Tris-HCl (pH 7.4).

For purification of SARS-CoV-2 GST-fused nsp10, the supernatant was loaded onto a GSTrap 4B column (5 mL, GE HealthCare). After washing with a washing buffer containing 50 mM Tris-HCl (pH 7.4), 150 mM NaCl and 10 mM β-mercaptoethanol, the GST-fused nsp10 was eluted by a buffer containing 50 mM Tris-HCl (pH 7.4), 150

mM NaCl, 10 mM β-mercaptoethanol and 10 mM glutathione. The GST-fused nsp10 was further purified by a gel filtration column (HiLoad 16/60 Superdex 200, GE HealthCare) pre-equilibrated with a buffer of 50 mM Tris-HCl (pH 7.4) and 150 mM NaCl.

Zinc ejection assays. The Zn-ejection agents, 2,2'-dithiobis(benzothiazole), cystamine dihydrochloride, 5,5'-dithiobis(2-nitrobenzoic acid) (DTNB), phenyl disulfide, 3-nitrobenzamide, tetraethylthiuram disulfide (disulfiram), 2,2'-dithiodipyridine, (±)alpha-lipoic acid, ebselen and cisplatin were purchased from Sigma-Aldrich (USA). The zinc-specific fluorophore FluoZinTM-3 (Invitrogen/Life Technologies) was used to monitor the release of zinc ions from SARS-CoV-2 PL^{pro} and GST-fused nsp10. The Zn-ejecting agents were dissolved in DMSO to a stock solution of 100 mM. SARS-CoV-2 His-tagged PL^{pro} (5 μM) and GST-fused nsp10 (5 μM) was respectively mixed with each Zn-ejecting agent (5 μM) and FluoZin-3 (1 μM) in a total reaction volume of 200 μl (50 mM Tris-HCl, pH7.4) at room temperature. Fluorescence emission was then measured by EnSpire Multilabel Plate Reader (Perkin Elmer, USA) at an excitation wavelength of 494 nm and emission wavelength of 516 nm.

IC_{50} measurement for disulfiram and ebselen on PL^{pro} activity. The fluorogenic peptide substrate Dabcy-FTLKGGAPTKVTE-Edans-NH2 synthesized by Kelowna international Scientific Inc. (Taiwan) was used to measure the SARS-CoV-2 PL^{pro} activity. PL^{pro} (0.5 μM) was incubated with 50 μM peptide substrate in the presence of disulfiram (0–144 μM) or ebselen (0–48 μM) in the reaction buffer containing 20 mM sodium phosphate (pH 7.0) at 30℃. Fluorescence intensity was monitored at 490 nm (emission) with the excitation wavelength at 340 nm by EnSpire Multilabel Plate Reader (Perkin Elmer, USA). The increase in fluorescence in the time

interval 100–400 s was calculated as the initial velocity, which was used to calculate IC_{50} by sigmoidal nonlinear regression logistic four parameter analysis using GraphPad Prim software.

Molecular mass measured by MALDI-TOF MASS. SARS-CoV-2 PL^{pro} (40 μM) was respectively mixed with disulfiram (200 μM) and ebselen (200 μM), and incubated at room temperature for 30 min. SARS-CoV-2 GST-fused nsp10 (2 mg/ml) was incubated with TEV protease (0.2 mg/ml) overnight at 4°C to remove GST, and the protein sample (20 μM) was then incubated with disulfiram (400 μM) and ebselen (100 μM) at room temperature for 1 hr, respectively. The protein samples with or without disulfiram/ebselen were then denatured by 0.1 % TFA and passed through a $ZipTip_{C18}$ desalting column (Millipore) before analysis by Bruker Daltonics-Autoflex III TOF/TOF mass spectrometer (Bremen). Mass-spectra analysis was performed in positive ion mode on a Bruker Autoflex III MALDI TOF/TOF mass spectrometer (Bremen, Germany) equipped with a 200 Hz SmartBeam Laser. Data acquisition and processing were done manually by using FlexControl 3.4 and Flex-Analysis 3.4 (Bruker Daltonik GmbH), respectively.

Tandem mass spectrometry. NanoLC−nanoESi-MS/MS analysis was performed on a Thermo UltiMate 3000 RSLCnano system connected to an Thermo Orbitrap Fusion mass spectrometer (Thermo Fisher Scientific, Bremen, Germany) equipped with a nanospray interface (New Objective, Woburn, MA). SARS-CoV-2 PL^{pro} (20 μM) was incubated with disulfiram (200μM) or ebselen (400 μM) at room temperature for 30 min. Disulfiram- and ebselen-treated PL^{pro} were dissolved in urea and then digested by trypsin. Peptide mixtures were loaded onto a 75 μm ID, 25 cm length PepMap C18 column (Thermo Fisher Scientific) packed with 2 μm particles with a pore width of 100 Å, and were separated using a segmented gradient in 60 min from 5% to 35% solvent

B (0.1% formic acid in acetonitrile) at a flow rate of 300 nl/min with a solvent A of 0.1% formic acid in water. The mass spectrometer was operated in the data-dependent mode. Briefly, survey scans of peptide precursors from 350 to 1600 m/z were performed at 120K resolution with a 2×10^5 ion count target. Tandem MS was performed by isolation window at 1.6 Da with the quadrupole, HCD fragmentation with normalized collision energy of 30, and MS^2 scan analysis at 30K resolution in the orbitrap. The MS^2 ion count target was set to 5×10^4 and the maximum injection time was 54 ms. Only those precursors with charge state 2–6 were sampled for MS^2. The instrument was run in top speed mode with 3 s cycles; the dynamic exclusion duration was set to 15 s with a 10 ppm tolerance around the selected precursor and its isotopes. Monoisotopic precursor selection was turned on. The raw data obtained from LC-MS/MS acquisition was processed using Proteome Discoverer (version 2.4; Thermo Scientific), searching file using Mascot search engine (v.2.7.0; Matrix Science, Boston, MA, USA) against the customized target proteins database. Search criteria used were trypsin digestion, allowing up to 2 missed cleavages, mass accuracy of 10 ppm for the parent ion and 0.02 Da for the fragment ions. Variable modifications set as oxidation (methionine), disulfiram (cysteine) and ebselen (cysteine). A decoy database search was performed. Identified peptides were filtered with 1% FDR. Modification sites and peptide sequence assignments contained in MASCOT search results were validated by manual confirmation from raw MS/MS data.

Cell-based assays. Vero E6 cells were pretreated with the disulfiram/ebselen and/or hydroxychloroquine (HCQ) at various concentrations for 1 hr at 37°C and then adsorbed with SARS-CoV-2 (TCDC#4) at MOI 0.005 (100 PFU/well) for 1 hr at 37°C. After virus adsorption, the cells were replenished with fresh medium and compounds at the indicated concentrations for 1-day incubation. The cells were fixed with 10%

formaldehyde, permeabilized with 0.5% Triton X-100, and stained with anti-SARS-CoV-2 N protein antibody (provided by Dr. An-Suei Yang, Genomic Research Center, Academia Sinica, Taiwan) and anti-human IgG-488 (green). The N protein expression was measured using a high-content image analysis system (Molecular Devices) and the average infection rate of no-drug treatment was set as 100%. The percentage of inhibition was calculated as 100% – infection rate. For cell viability test, Vero E6 cells were treated with each compound for 24 hrs and cell viability was determined by Cell Counting Kit-8 (CCK-8). IC_{50} and CC_{50} were calculated by Prism software.

References

1. McDonnell, N. B.; Guzman, R. N. D.; Rice, W. G.; Turpin, J. A.; Summers, M. F., Zinc Ejection as a New Rationale for the Use of Cystamine and Related Disulfide-Containing Antiviral Agents in the Treatment of AIDS. *J. Med. Chem.* **1997**, 40, 1969-1976.

2. Lenstra, D. C.; Temimi, A. H. K. A.; Mecinovic, J., Inhibition of histone lysine methyltransferases G9a and GLP by ejection of structural Zn(II). *Bioorg. & Med. Chem. Letters* **2018**, 28, 1234-1238.

3. Mateo, J.; Ong, M.; Tan, D. S. P.; Gonzalez, M. A.; Bono, J. S. d., Appraising iniparib, the PARP inhibitor that never was—what must we learn? *Nat. Rev. Clin. Oncol.* **2013**, 10, 688–696.

4. Sekirnik, R.; Rose, N. R.; Thalhammer, A.; Seden, P. T.; Mecinovic, J.; Schofield, C. J., Inhibition of the histone lysine demethylase JMJD2A by ejection of structural Zn(ii). *Chem. Commun.* **2009**, 6376-6378.

5. McDonald, I. K.; Thornton, J. M., Satisfying hydrogen bonding potential in proteins. *J. Mol. Biol.* **1994**, 238, 777-793.

6. Canutescu, A. A.; Shelenkov, A. A.; Dunbrack, R. L., Jr., A graph-theory algorithm for rapid protein side-chain prediction. *Protein Sci.* **2003**, 12, 2001-2014.

7. Webb, B.; Sali, A., Comparative Protein Structure Modeling Using MODELLER. *Curr. Protoc. Bioinformatics* **2016**, 54, 5.6.1-5.6.37.
